# Thermal Performance and Stability of CO_2_‑Rich Biogas Combustion in a Heat-Recuperated Radiant Porous Burner

**DOI:** 10.1021/acsomega.6c03415

**Published:** 2026-07-14

**Authors:** José Alexandre de Campos, Fabio Bongoski, Roberto Wolf Francisco

**Affiliations:** Mechanical Engineering Department, 74382 Universidade do Estado de Santa Catarina, Rua Paulo Malschitzki 200, Joinville 89219-710, Santa Catarina, Brazil

## Abstract

Low-calorific-value biogas with high CO_2_ content presents significant challenges for conventional combustion systems due to reduced flame stability, lower reaction temperatures and diminished thermal efficiency. Developing efficient technologies capable of directly utilizing CO_2_-rich biogas without costly upgrading processes is therefore essential for expanding its use in renewable heat generation. This study presents a combined experimental and numerical investigation of the effects of CO_2_-rich biogas composition and reactant preheating on the performance of a radiant porous burner equipped with an integrated heat recovery system. Biogas was simulated using CH_4_/CO_2_ mixtures containing up to 65 vol % CO_2_, and the burner was operated under ultralean conditions at an equivalence ratio of 0.50. The system consists of a two-layer porous medium and a radially integrated stainless-steel coil heat exchanger designed to enhance internal heat recirculation and promote reactant preheating. Flame stability limits, temperature profiles, outlet surface temperature, radiative efficiency and pollutant emissions were experimentally evaluated. In parallel, a thermodynamic model based on a global energy balance was applied to quantify energy losses relative to an ideal adiabatic system, while detailed kinetic simulations using the GRI-Mech 3.0 were conducted to assess adiabatic laminar flame speed and temperature. Increasing the CO_2_ concentration reduced the burner stability range and shifted the reaction zone toward the outlet due to lower flame temperatures and slower reaction kinetics. Reactant preheating increased the inlet temperature to approximately 100 °C and expanded the stability range by up to 75% for the 50% CH_4_/50% CO_2_ mixture, while also reducing CO emissions. The maximum CO emission index was 0.082 g kg^–1^, whereas NO emissions remained below detection limits. Energy losses ranged from 13 to 17%, with higher flow velocities primarily increasing exhaust-gas losses.

## Graphical abstract

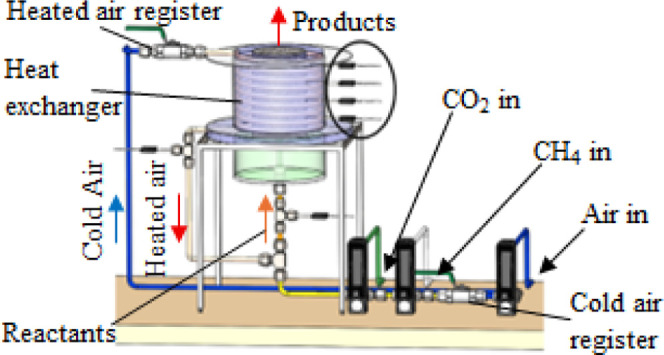

## 1 Introduction

Biogas is produced through the anaerobic decomposition of organic substrates, and its composition depends strongly on feedstock characteristics and digestion conditions. It consists mainly of methane (CH_4_) and carbon dioxide (CO_2_), along with smaller fractions of nitrogen, oxygen, and trace contaminants such as sulfur compounds, siloxanes, and ammonia.^1^ Typical methane contents range from 49 to 77%, while CO_2_ concentrations vary between 22 and 44%.^1,2^ As a result, its heating value is significantly lower than that of pure methane,^3^ which directly affects combustion behavior and limits the performance of conventional combustion systems.

Although biogas upgrading technologies are well established for producing biomethane suitable for grid injection and transportation applications,^4^ they require additional capital investment, energy consumption, maintenance, and operational complexity.^5^ These requirements may limit their economic feasibility, particularly in decentralized small-scale systems. In contrast, the direct use of raw biogas for on-site thermal applications avoids upgrading costs but requires combustion systems capable of operating efficiently with low-energy-density and highly diluted fuels. This challenge motivates the present investigation of porous burners with integrated heat recovery.

Porous media combustion has gained increasing attention due to its ability to efficiently utilize low-calorific-value fuels across a wide range of applications^6–11^ and to enhance combustion performance under challenging conditions.^12–14^ The porous matrix promotes internal heat recirculation through conduction and radiation, transferring energy from the high-temperature combustion zone to the incoming reactants. This internal energy feedback leads to reactant preheating, increases reaction-zone temperatures, promotes earlier ignition, and accelerates chemical reaction rates, enabling stable combustion under ultralean and highly diluted conditions.^15–17^

The combustion behavior in porous burners is strongly influenced by fuel composition, burner geometry and porous material properties, which directly affect flame stabilization, thermal efficiency and heat transfer mechanisms.^18–21^ In particular, variations in the CH_4_/CO_2_ ratio alter the fuel heating value, adiabatic flame temperature and laminar flame speed. Increasing CO_2_ concentration reduces mixture reactivity due to thermal dilution and increased heat capacity, which may shift the flame front, narrow stability limits, and reduce radiative output.

Gao et al.^22^ investigated combustion in a two-layer porous burner packed with alumina spheres operating with methane diluted by CO_2_, N_2_, and Ar, analyzing the influence of inert gas type on burner performance. In their study, CO_2_ concentrations ranged from 25% to 40%, while the equivalence ratio varied between 0.75 and 0.90. Similarly, most previous studies have focused on biogas mixtures with CO_2_ concentrations below approximately 40%,^23^ whereas investigations at higher dilution levels remain limited.

In addition to fuel composition effects, operational strategies such as reactant preheating play a key role in extending flame stability limits. Increasing reactant temperature broadens the stability range and enables combustion under extremely lean conditions. This behavior has been demonstrated in porous burners specifically designed to enhance internal heat recirculation. Song et al.,^15^ for example, investigated an annular porous burner that promoted internal heat feedback, allowing stable combustion of CH_4_/N_2_ mixtures under extended lean conditions. Similarly, Vandadi et al.^16^ numerically analyzed a superadiabatic radiant porous burner incorporating an external preheater and radiation corridors, where heat recovered from exhaust gases increased the inlet temperature and improved overall thermal performance.

Complementary experimental studies have also demonstrated the advantages of porous radiant burners for low-calorific-value fuels. Double-layer burners composed of SiC and Al_2_O_3_ matrices have been shown to sustain stable lean combustion while significantly reducing pollutant emissions compared to conventional systems, achieving substantial reductions in CO and NO_*x*_ emissions along with improved combustion efficiency within operating ranges of 5–10 kW and equivalence ratios between 0.75 and 0.97.^24^

In parallel, numerical investigations of biogas diffusion flames have highlighted the strong influence of fuel composition on flame structure and emissions.^25^ Increasing CO_2_ concentration reduces flame temperature and radical concentrations due to both thermal and chemical effects, leading to lower NO formation, whereas hydrogen enrichment enhances mixture reactivity and increases emission indices. Additional studies have explored strategies to improve ignition and stability in porous media systems, including advanced ignition techniques capable of rapidly establishing a flame within the porous matrix without significantly altering its structure.^26^ These approaches have demonstrated stable combustion of methane and biogas mixtures with CO_2_ concentrations between 15% and 40% under lean conditions, with fast thermal response and low pollutant emissions.

Moreover, externally preheated air supplied by auxiliary systems, such as solar-assisted heaters, has been used to support biogas combustion in porous radiant burners.^27^ In these configurations, preheating enabled stable operation at equivalence ratios between 0.70 and 0.91, with CO_2_ concentrations up to approximately 35%, while achieving radiation efficiencies between 15% and 48% and maintaining low NO_*x*_ emissions. Data-driven approaches have also been applied to predict burner performance, showing good agreement with experimental results.

Despite these advances, most existing studies remain limited to moderate CO_2_ dilution levels,^28^ near-stoichiometric or moderately lean conditions, or rely on external heating systems to achieve reactant preheating. Consequently, the combined influence of reactant preheating and high CO_2_ dilution is still not fully understood. This limitation is particularly relevant for CO_2_ concentrations above 50 vol % under ultralean conditions (Ø ≈ 0.5) and moderate preheating levels (80–150 °C), where their impact on flame stability, reaction-zone temperature, radiative efficiency, and pollutant emissions has not been systematically investigated.

Although reactant preheating, especially when implemented through integrated heat recovery, can enhance flame stability and partially compensate for the reduced reactivity of CO_2_-rich mixtures, comprehensive experimental and numerical studies addressing these combined effects under highly diluted conditions remain scarce.

Therefore, the present study aims to experimentally and numerically investigate the effects of CO_2_-rich biogas composition and reactant preheating, achieved through an integrated heat exchanger, on the performance of a radiant porous burner. From a numerical standpoint, the burner is analyzed using a thermodynamic framework based on a global energy balance, assuming an adiabatic system, complete combustion represented by a single-step global reaction, and thermal equilibrium between the gas and solid phases. This approach enables the quantification of energy losses and their impact on radiative efficiency through comparison between experimental results and an idealized model. In addition, detailed simulations using the GRI-Mech 3.0 ^29^ chemical kinetic mechanism are conducted based on the experimental conditions, allowing a more physically consistent assessment of flame stabilization and heat transfer in CO_2_-diluted porous media combustion.

This study contributes to the literature by (i) experimentally investigating biogas combustion with high CO_2_ concentrations beyond the commonly studied range, focusing on burner operability and flammability limits; (ii) proposing a novel burner configuration incorporating a radially integrated stainless-steel heat exchanger for reactant preheating; (iii) providing a comprehensive performance evaluation of a radiant porous burner operating under ultralean and highly diluted conditions, including reaction temperature, radiative efficiency, and emissions; and (iv) elucidating the combined effects of reactant preheating and CO_2_ dilution on adiabatic flame speed and temperature, highlighting the role of heat recovery in the combustion of low-energy-density fuels. To isolate these effects, experiments and simulations were conducted by independently varying fuel composition and inlet temperature while maintaining constant equivalence ratio and operating conditions.

## 2 Methodology

### 2.1 Experimental Setup

Figure 1 shows the schematic diagram of the experimental apparatus used. The fuels used in the experiments were methane and biogas, with biogas being simulated from a mixture of different proportions of CH_4_ and CO_2_. Biogas was simplified as a CH_4_/CO_2_ mixture to isolate the dominant effects of CO_2_ dilution, while minor contaminants such as H_2_S, siloxanes, and moisture were neglected due to their relatively low concentrations and secondary influence on global combustion behavior.

**Figure 1:**
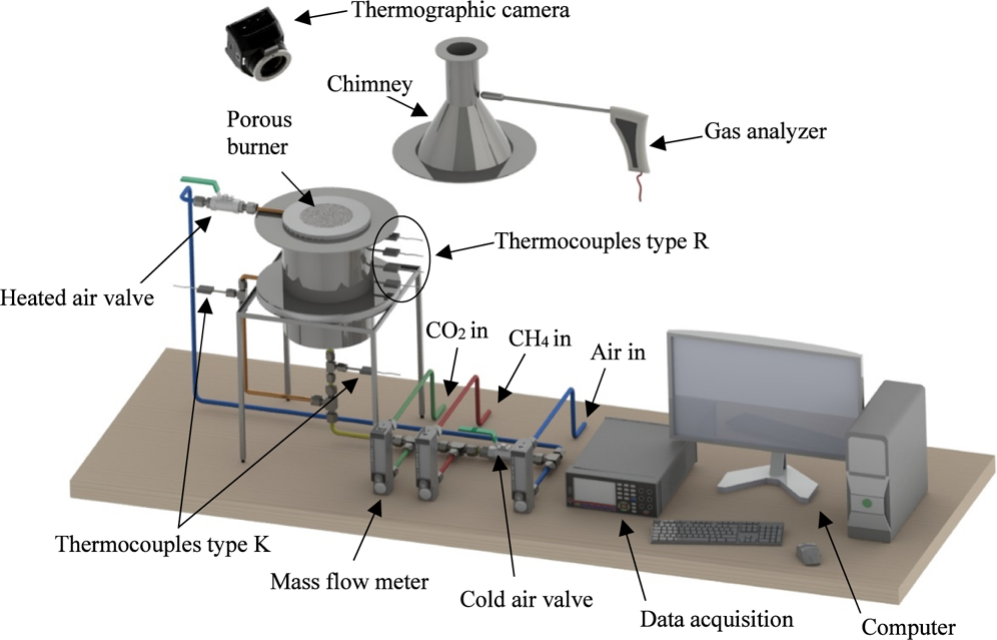
Schematic diagram of the experimental apparatus for burner analysis.

The gases are supplied to the mass flow controllers OMEGA brand, model FMA-2608A, with a flow range of 0 to 20 SLPM for CH_4_, and OMEGA model FMA-2609A for CO_2_, with a flow range of 0 to 50 SLPM. The air flow is controlled by means of a fine adjustment valve and monitored with the aid of an OMEGA brand flow meter, model FMA-A2323, with a flow range of 0 to 100 SLPM. The flow meters were factory-calibrated according to the manufacturer’s specifications.

The instruments provide the flow rate in Standard Liters per Minute (SLPM), corresponding to standard reference conditions of 25 °C and 1 atm. A pressure regulator was installed upstream of the flow meter to ensure stable operating conditions throughout the experiments. During the test runs, pressure fluctuations at the flow meter inlet remained below ±0.01 bar, while air temperature variations were approximately ±1 °C around room temperature. Since the flow rate is reported in SLPM, these small variations in actual operating conditions did not affect the indicated flow rate, which was continuously monitored and maintained at the prescribed set point. Therefore, no additional corrections for temperature or pressure were applied. The uncertainties associated with the flow measurements were accounted for in the overall error analysis.

During the tests performed with reactant preheating, the cold air valve was kept closed while the hot air valve was opened. Under this configuration, the airflow was routed through the heat exchanger integrated into the burner body, thereby promoting adequate reactant preheating prior to the reaction stage. In contrast, during the tests conducted without reactant preheating, the hot air valve remained closed and the cold air valve was opened, as illustrated in Figure 1.

The porous burner used in the experimental analysis is of the two-layer type, similar to that used by,^30^ but with a longer stabilization region, consisting of five silicon carbide (SiC) ceramic foams, with an average diameter of 70 mm and an average height of 21.5 mm, as shown in Figure 2.

**Figure 2:**
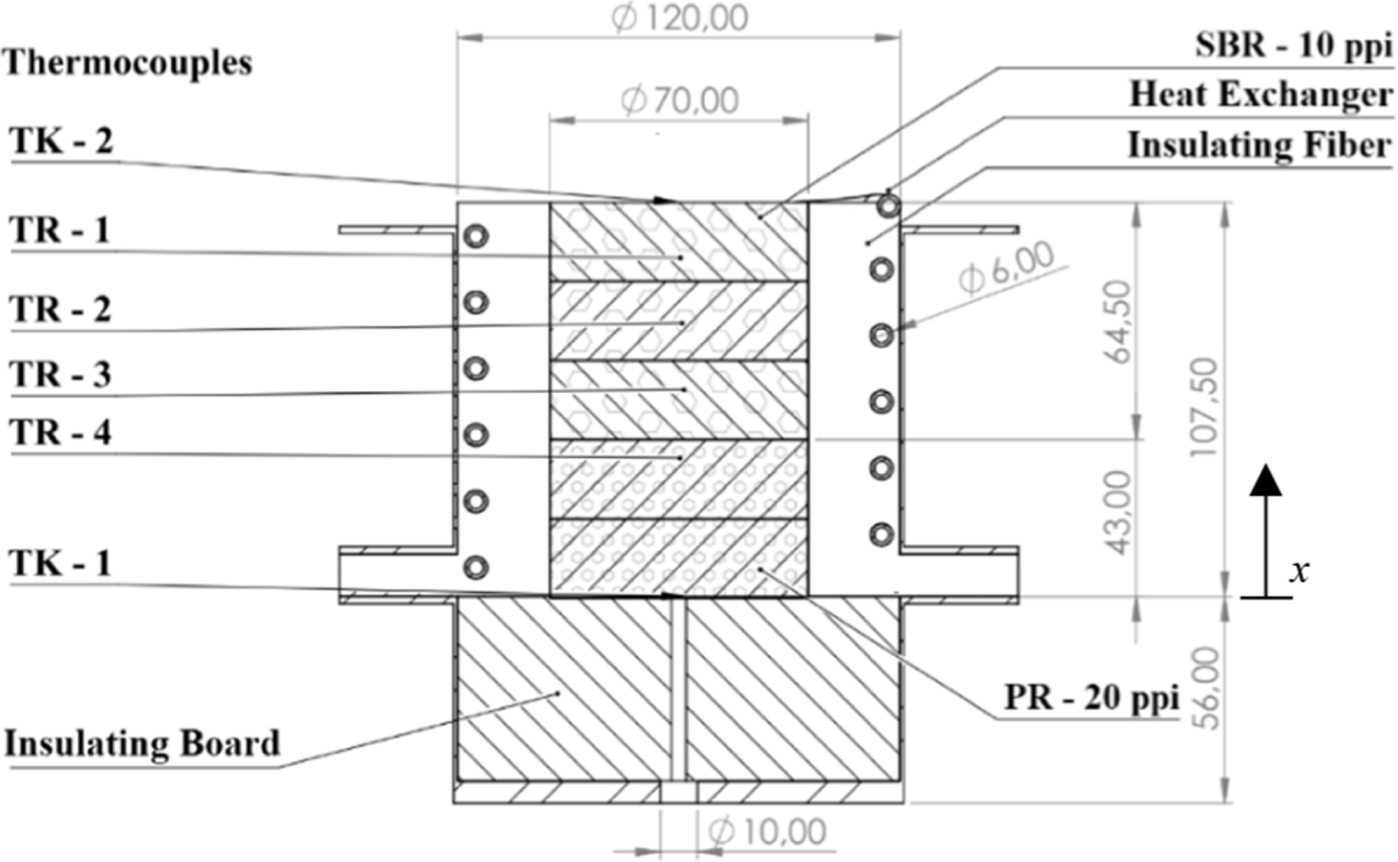
Schematic drawing of the porous burner.

The burner’s first layer is the preheating region (PR), located above the injection plate and composed of two 20 ppi ceramic foams. The higher porosity enhances reactant mixing and helps prevent flashback, which could lead to flame propagation toward the injection plate and potential explosion. The second layer is the stable combustion region (SBR), positioned downstream and made of three 10 ppi ceramic foams. The porous medium is surrounded by a ∼20 mm fiberglass insulation layer. The heat exchanger is a 6-turn coil of 304L stainless steel tubing (6.0 mm outer diameter, 1.0 mm wall thickness, 2.0 m length, and 120.0 mm coil diameter).

The monitoring system includes thermocouples, a data acquisition system, a thermographic camera, and a gas analyzer. Four type R thermocouples are placed along the burner axis in the combustion region, while type K thermocouples are installed at the base, surface, and reactant lines. The thermocouples were installed along the burner axis to characterize the axial temperature distribution and indicate the region close to the flame front within the porous medium. Therefore, the highest recorded temperature corresponds to the maximum value measured at the instrumented locations and should not be interpreted as the absolute local peak temperature or the exact flame-front position, both of which may occur between adjacent thermocouples.

Three type K thermocouples measure reactant temperatures: two at the heat exchanger inlet and outlet and one at the burner inlet. All thermocouples are connected to a Keysight DAQ970A system operated with BenchVue software to evaluate safe and stable operation and temperature profiles. Surface temperatures are also measured with a FLIR T650sc thermographic camera and analyzed in FLIR Thermal Studio.

CO and NO emissions are measured using a TESTO 350 gas analyzer via a removable exhaust stack, with the probe placed at the duct center and data recorded using Easy Emission software. Measurement uncertainties for all devices are listed in Table 1.

**Table 1:** Measurement Uncertainty of the Experimental Apparatus Components

measurement instrument	uncertainty
CH_4_ mass flow controller	±(0.8% of reading +0.2% of full scale)
CO_2_ mass flow controller	±(0.8% of reading +0.2% of full scale)
Air mass flow meter	±1.0% of full scale
Thermographic camera	±(1.0°C ± 1.0% of reading)
Type K thermocouple	±2.2 °C or 0.75%
Type R thermocouple	±1.5 °C or 0.25%
NO emission	±5 ppm (0–99 ppm)/ ± 5% (100–1999 ppm)
CO emission	±10 ppm (0–199 ppm)/ ± 5% (200–2000 ppm)

### 2.2 Experimental Parameters

Equation 1 presents the expression used to calculate the equivalence ratio Ø as a function of the mass flow rates of air and fuel supplied to the burner

1 $$Ø=\frac{{\left({ṁ}_{fuel}/{ṁ}_{a\mathrm{ir}}\right)}_{app}}{{\left({ṁ}_{fuel}/{ṁ}_{a\mathrm{ir}}\right)}_{st}}$$

where ${ṁ}_{fuel}$ and ${ṁ}_{air}$ are the mass flow rates of fuel and air, respectively. The subscripts app and st denote the applied and stoichiometric ratios. For a planar and one-dimensional flame, the mean flow velocity (*u*_0_) can be calculated as the ratio between the volumetric flow rate of the reactants (V̇) and the burner cross-sectional area (*A*_*s*_), as given by eq 2

2 $$u_{0}=\frac{\mathrm{V̇}}{A_{s}}$$

The thermal power supplied to the burner, ${Ṡ}_{r}$ (kW), can be calculated according to the following eq 3

3 $${Ṡ}_{r}={ṁ}_{fuel}\cdot LHV$$

where the term LHV (kJ/kg) represents the lower heating value of the fuel. Radiation efficiency (η_rad_) is one of the main parameters used to evaluate the performance of a radiant porous burner. It is defined as the ratio between the radiation heat transfer rate at the burner outlet surface (*q*_rad_) and the thermal power supplied ( ${Ṡ}_{r}$ ) and can be expressed as^22,23^

4 $$n_{rad}=\frac{q_{rad}}{{Ṡ}_{r}}=\frac{\varepsilon \sigma A_{s}\left(T_{\sup}^{4}-T_{\infty }^{4}\right)}{{ṁ}_{fuel}\cdot LHV}$$

where ε is the emissivity of the porous medium, σ is the Stefan–Boltzmann constant (kW·m^–2^·K^–4^) and *T* (K) is the temperature. The subscripts sup and ∞ refer to the burner surface and the environment, respectively. The emissivity was assumed to be equal to 0.99.

Table 2 presents the maximum uncertainties of the evaluated parameters, estimated through standard error propagation based on eqs 1 to 4, using the uncertainties of the measured variables listed in Table 1.

**Table 2:** Maximum Measurement Uncertainties

parameter	uncertainty
Mean flow velocity (*u*_0_)	3.7 cm/s
Equivalence ratio (Ø)	±0.11
Thermal power supplied ( ${Ṡ}_{r}$ )	±0.1 kW
Radiative efficiency (η_rad_)	±5.1%

### 2.3 Experimental Procedure

The porous burner is ignited at the gas outlet surface by adjusting the initial ignition point. After the flame enters the porous medium, temperatures measured by Type R thermocouples are monitored via the data acquisition system, and the flame front is identified as the region of maximum temperature. The initial configuration is maintained until the temperature at the burner base reaches 900 °C. Then, the first operating condition is established by adjusting the mass flow rates of air, methane, and carbon dioxide, according to the mean flow velocity, equivalence ratio, and fuel composition. Steady-state conditions were assumed when temperatures within the porous medium remained constant for at least 20 min. Under these conditions, the stability and uniformity of the preheated air were ensured by monitoring temperatures at the heat exchanger outlet and burner inlet, which showed minimal variation. The coil geometry and associated flow residence time promoted effective thermal homogenization prior to injection.

The upper stability limit (USL), or blowout limit, occurs when the porous medium temperature exceeds 1350 °C or when high reactant flow rates push the flame front out of the porous medium, making it visible at the burner surface. This temperature limit was imposed to avoid material degradation. The lower stability limit (LSL) is reached when the temperature at the injection plate thermocouple exceeds 1000 °C, indicating potential flashback.

After reaching steady-state conditions, the burner surface temperatures are measured using a thermal imaging camera, and the images are recorded for analysis. Subsequently, pollutant emissions are measured with a gas analyzer through a removable conical chimney.

### 2.4 Thermodynamic Approach

Figure 3 presents a schematic representation of the radiant porous burner evaluated in the present study. The dashed line indicates the global control volume applied to the burner.

**Figure 3:**
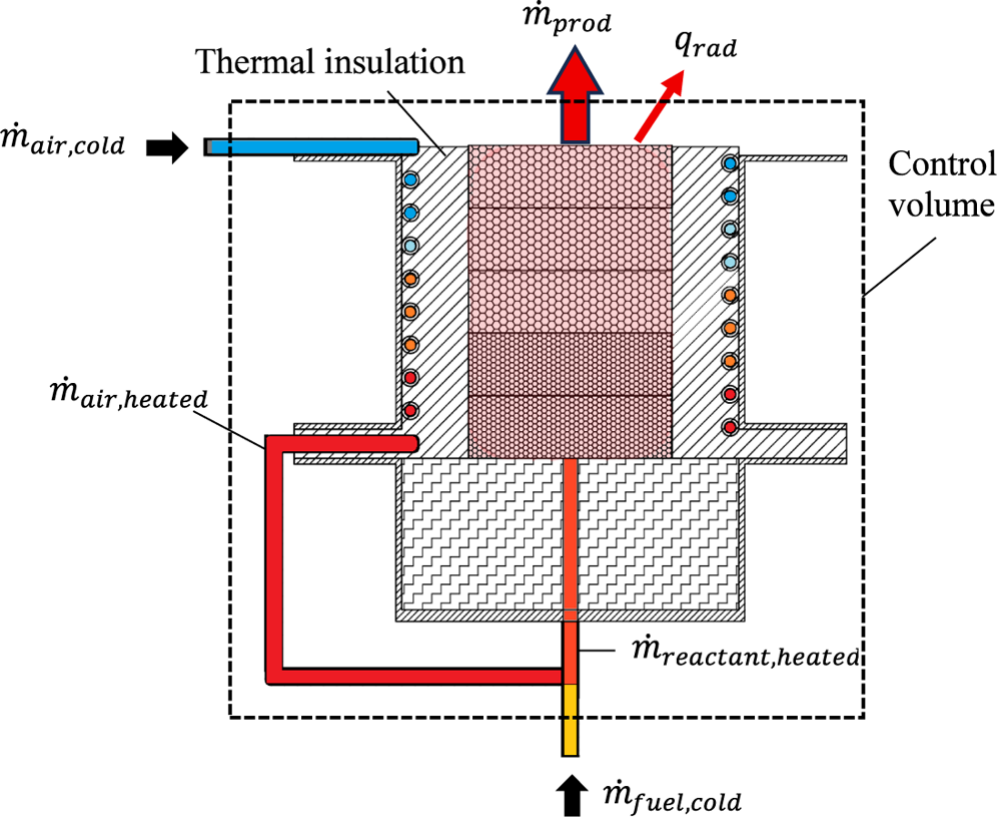
Schematic drawing of the analytical model applied in the work.

In this model, heat losses through the burner walls, the effects of residence time and chemical species dissociation in the combustion products were neglected, allowing the determination of the maximum theoretical reaction temperature (*T*_*r*_) and maximum radiative efficiency. Therefore, the global energy balance results in the following equation

5 $${ṁ}_{fuel}LHV+{ṁ}_{air}{\Delta h}_{sens,air}+{ṁ}_{fuel}{\Delta h}_{sens,fuel}={ṁ}_{prod}{\Delta h}_{sens,prod}+\varepsilon \sigma A_{s}\left(T_{r}^{4}-T_{\infty }^{4}\right)$$

the terms Δ*h*_sens,air_ and Δ*h*_sens,fuel_ represent the sensible enthalpy of air and fuel, respectively, which can be neglected since both enter the burner at ambient temperature (298 K). The term Δ*h*_sens,prod_ represents the sensible enthalpy of the combustion products, calculated assuming complete combustion represented by a single-step global reaction, containing CO_2_, N_2_, H_2_O e O_2_ at reaction temperature (*T*_*r*_), as shown in eq 6

6 $$\alpha CH_{4}+\left(1-\alpha \right)CO_{2}+n_{air}\left(O_{2}+3.76N_{2}\right)\to 1CO_{2}+\left(2\cdot \alpha \right)H_{2}O+\left(n_{air}\cdot 3.76\right)N_{2}+\left(n_{air}-2\cdot \alpha \right)O_{2}$$

This equation is valid only for lean mixtures (Ø ≤ 1.0). Assuming thermal equilibrium between the solid and gaseous phases, the radiative heat transfer rate and the sensible enthalpy of the combustion products must be evaluated at the reaction temperature (*T*_*r*_).

Thus, the input parameters of the model were the same as those used in the experiments, namely the equivalence ratio and the mean flow velocity, which allowed the calculation of the fuel and air mass flow rates. Subsequently, by applying eq 5 together with the global combustion reaction defined in eq 6, the reaction temperature (*T*_*r*_) can be determined. Based on this temperature, the radiative heat transfer rate, the radiative efficiency, and the energy carried by the exhaust gases can then be evaluated.

## 3 Results and Discussion

All analyses were conducted at an equivalence ratio of 0.5 and under atmospheric pressure. Table 3 summarizes the fuel compositions employed, the lower heating value (LHV) of each mixture, and the corresponding adiabatic flame temperature without preheating (298 K). Reactant preheating was evaluated using pure methane (100% CH_4_) and simulated biogas composed of 50% CH_4_ + 50% CO_2_ as fuels.

**Table 3:** Fuel Mixture Compositions, Lower Heating Values, and Corresponding Adiabatic Flame Temperatures without Preheating (298 K)

fuel mixture (% vol.)	LHV (kJ/kg)	adiabatic flame temperature (°C)^a^
100% CH_4_	50045	1205.2
75% CH_4_ + 25% CO_2_	26141	1180.6
50% CH_4_ + 50% CO_2_	13369	1132.1
35% CH_4_ + 65% CO_2_	8211	1075.6

a Estimated using the GRI-Mech 3.0 chemical kinetic mechanism, as described in Section 3.5.

### 3.1 Flame Stability Range

Figure 4 shows the flame stability ranges for the different fuels tested as a function of the CH_4_ concentration in the mixture, varying from 35% to 100% by volume, at an equivalence ratio of 0.5. The stability range obtained without reactant preheating through the coil is shown in black markers, while the results with reactant preheating are indicated in red markers. The dotted lines indicate the stable combustion limits for the burner with preheating (red line) and without preheating (black line). The areas between the lines indicates the stable combustion region for the burner operating at different conditions.

**Figure 4:**
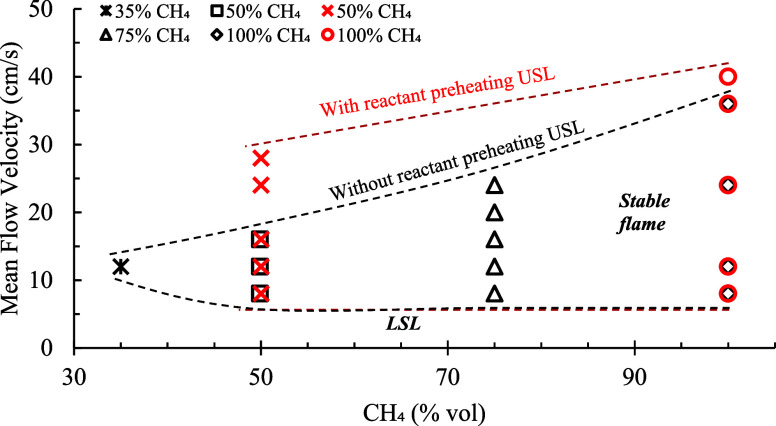
Flame stability range as a function of CH_4_ concentration, without preheating (black markers) and with preheating (red markers) for Ø = 0.5.

A reduction in the stable operating range of the burner can be observed with increasing CO_2_ concentration in the biogas mixture. First, considering the tests performed without reactant preheating, the burner operated stably with pure methane over mean flow velocities ranging from 8 cm/s, corresponding to the lower stability limit, up to 36 cm/s at the upper limit. This range corresponds to a thermal power between 0.5 kW and 2.2 kW. For a biogas mixture containing 50% CH_4_ + 50% CO_2_, the stable operating range was reduced to flow velocities between 8 and 16 cm/s, corresponding to thermal powers from 0.48 kW to 0.96 kW. This reduction results from the lower reaction-zone temperature caused by CO_2_ dilution, which affects reaction rates and the adiabatic laminar flame speed.^31^ The fuel mixture with the lowest heating value that could be stably operated consisted of 35% CH_4_ and 65% CO_2_ (8211 kJ/kg), which represents the operational flammability limit of the burner under the investigated conditions. Biogas mixtures with CO_2_ concentrations higher than 65% were unable to sustain a stable flame in the burner.

The effects of reactant preheating are shown by the red markers in Figure 4, where a clear expansion of the burner stable operating range is observed. The lower stability limit remained unchanged for both fuels, pure methane and biogas (50% CH_4_ + 50% CO_2_), at a minimum mean flow velocity of 8 cm/s. In contrast, the upper stability limit increased significantly with preheating. For the biogas mixture, it rose from 16 to 28 cm/s, corresponding to a thermal power of 1.7 kW, representing an expansion of approximately 75%. For pure methane, the maximum flow velocity increased from 36 to 40 cm/s (2.5 kW), corresponding to an increase of about 11%.

### 3.2 Effects of Composition and Preheating on the Temperature Profile

Figure 5 shows the temperatures measured in the porous medium as a function of axial position along the burner length for CH_4_ concentrations ranging from 35% to 100%, without preheating, while maintaining a constant mean flow velocity of 12 cm/s and an equivalence ratio of 0.5.

**Figure 5:**
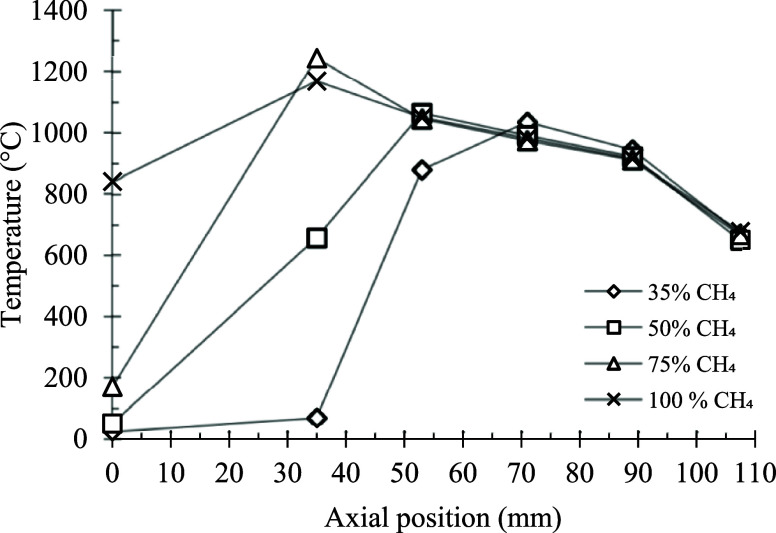
Temperatures measured in the porous medium as a function of axial position along the burner length, for biogas mixtures with CH_4_ concentrations between 35% and 100%, without preheating, *u*_0_ = 12 cm/s and Ø = 0.5.

The mixture containing pure CH_4_ exhibited the highest reaction temperature, on the order of 1244 °C. Furthermore, analysis of the peak temperatures recorded for each fuel mixture indicates that decreasing the CH_4_ concentration leads to a displacement of the flame front toward the burner outlet surface. This behavior is primarily associated with the reduced reaction temperature and adiabatic laminar flame speed resulting from CO_2_ dilution, as well as the slightly lower thermal power applied (approximately 5% reduction). The decrease in flame speed modifies the position of the reaction zone, which is governed by the thermochemical balance between the mean flow velocity and the laminar flame speed. As a result, with the reduction in methane concentration from 100% to 35%, the maximum temperature decreased from 1244 to 1035 °C, which corresponds to a reduction of approximately 16%.

Another observation is that, irrespective of the CO_2_ concentration, the temperatures measured at the burner outlet surface remained nearly constant, around 650 °C. This behavior is governed by the overall heat transfer balance, increasing CO_2_ concentration reduces the thermal power supplied to the burner and lowers the reaction temperature, which shifts the flame front toward the outlet. At the same time, the lower reaction temperature reduces heat losses due to smaller temperature gradients and a reduced effective heated region. As a result, the decrease in heat release is partially compensated by lower heat losses, leading to similar surface temperatures. This behavior is advantageous, as it indicates that even under significant variations in the CO_2_/CH_4_ ratio, such as those resulting from fluctuations in biogas composition, the radiative heat flux emitted from the burner surface remains approximately constant.

Table 4 presents the air temperatures measured at the inlet and outlet of the heat exchanger, as well as the reactant temperatures at the burner inlet after premixing with the fuel gas. The analysis with reactant preheating was carried out for pure methane and for a 50% CH_4_ + 50% CO_2_ mixture at an equivalence ratio of Ø = 0.5.

**Table 4:** Air Temperature at the Inlet and Outlet of the Heat Exchanger and Reactant Temperature at an Equivalence Ratio of Ø = 0.5

temperatures (°C)	fuel					fuel				
	50% CH_4_ + 50% CO_2_					100% CH_4_				
	*u*_0_ (cm/s)					*u*_0_ (cm/s)				
	8	12	16	24	28	8	12	24	36	40
	${Ṡ}_{r}$ (kW)					${Ṡ}_{r}$ (kW)				
	0.48	0.73	0.97	1.45	1.69	0.51	0.76	1.52	2.29	2.54
Air at the heat exchanger inlet	25.8	25.6	25.5	24.2	24.9	27.7	27.4	25.5	25.7	25.8
Air at the heat exchanger outlet	172.2	185.2	178.0	159.0	148.7	183.3	209.5	209.6	197.3	189.0
Reactants	83.4	102.8	108.3	111.2	108.2	87.2	113.1	142.5	148.1	146.0

With the use of the heat exchanger, the air temperature was increased to 185 °C for biogas mixture and to 209 °C for pure methane, resulting in reactant preheating to temperatures in the range of approximately 83 to 148 °C, depending on the fuel composition and the mean flow velocity.

Figure 6 shows the temperature profiles measured along the porous medium as a function of axial position along the burner length for (a) pure methane and (b) a biogas mixture (50% CH_4_ + 50% CO_2_), at an equivalence ratio of 0.5. The results are presented for conditions without reactant preheating (black markers) and with preheating (red markers). The position of the reaction zone can be inferred from the location of the thermocouple that records the maximum temperature. Each curve corresponds to a different mean inlet velocity (*u*_0_), expressed in cm/s.

**Figure 6:**
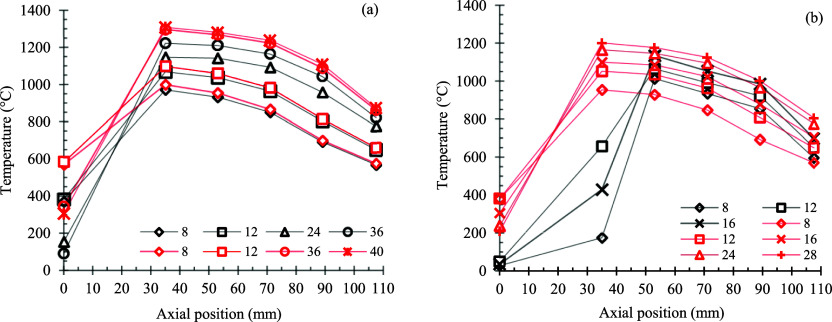
Temperature profiles inside the porous medium as a function of axial position for (a) pure methane and (b) 50% CH_4_ + 50% CO_2_, at different mean flow velocities (*u*_0_, cm/s) and Ø = 0.5, with (red markers) and without (black markers) reactant preheating.

Initially, the temperature profiles for pure methane (Figure 6a) without reactant preheating showed that the reaction temperature increased from 971 to 1222 °C as the mean flow velocity rose from 8 to 36 cm/s, due to the higher thermal power supplied to the burner. For all investigated power levels, the flame front remained close to the interface between the porous layers (∼35 mm), consistent with the stabilizing effect of the lower-porosity layer reported in the literature.^32^ Although the peak temperature position remained nearly unchanged, temperatures near the burner base decreased with increasing flow velocity.

When reactant preheating was applied, an overall temperature increase was observed along the axial position, with the most significant differences occurring near the burner base, where temperatures rose by approximately 200 °C on average. In the reaction zone, the effect was less pronounced: at 8 cm/s, the maximum temperature increased from 971.1 to 998.7 °C, while at 36 cm/s it increased from 1222.0 to 1296.1 °C. The highest temperature recorded was 1308.1 °C at 40 cm/s under preheated conditions.

Moreover, Figure 6b shows that, for all evaluated mean flow velocities, preheating shifted the flame front upstream, from approximately 53 mm to 35 mm, and increased the base temperatures by more than 180 °C, reflecting the intensification of reaction rates associated with higher flame temperatures.

### 3.3 Radiative Efficiency

Figure 7 presents (a) the temperatures measured at the outlet surface of the porous burner and (b) radiative efficiency as a function of the mean flow velocity and for different fuel mixtures, with reactant preheating (red markers) and without reactant preheating (black markers), while maintaining a constant equivalence ratio of Ø = 0.50.

**Figure 7:**
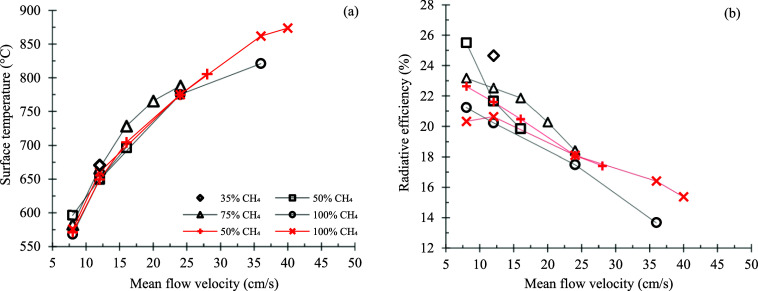
(a) Outlet surface temperature of the porous burner and (b) radiative efficiency as a function of the mean flow velocity for different fuel compositions, under preheated (red markers) and nonpreheated (black markers) reactant conditions at Ø = 0.50.

Under nonpreheated conditions (black markers) and at a constant mean flow velocity (*u*_0_ = 12 cm/s), decreasing the CH_4_ concentration from 100% to 35%, with a corresponding increase in CO_2_ and reduction in the lower heating value from 50.0 to 8.2 MJ/kg, led to a slight increase in outlet surface temperature from 649 to 670 °C (∼3%). Although the supplied thermal power decreased by 8.5%, this behavior is attributed to reduced heat losses.

With reactant preheating (red markers), increasing the inlet temperature had no significant effect on the outlet surface temperature for either fuel mixture.

Regarding radiative efficiency (Figure 7b), under nonpreheated conditions and at 12 cm/s, reducing the CH_4_ content from 100% to 35% increased efficiency from 20.2% to 24.6%, despite a decrease in thermal power (0.76 to 0.70 kW). The lower reaction temperature shifts the flame front downstream due to reduced adiabatic flame speed, maintaining nearly constant surface temperature while decreasing the heated wall area and associated heat losses, thereby enhancing radiative efficiency.

With preheating, at 8 cm/s both mixtures showed slightly higher efficiency without preheating. At higher velocities, efficiencies were similar or marginally higher with preheating. Since preheating does not alter the supplied thermal power, its effect is mainly associated with increased reaction temperature and flame position. However, variations in radiative efficiency under both conditions remained within experimental uncertainty.

For all mixtures, radiative efficiency decreased with increasing mean flow velocity, consistent with previous findings,^30^ due to higher thermal input, shorter residence time, increased wall heat losses, and greater energy transport by combustion products at the outlet surface.

However, in order to better understand the influence of each of these effects, the burner was numerically evaluated using a thermodynamic approach to quantify the different energy contributions involved in the system. For the mixture composed of 50% CO_2_ + 50% CH_4_, and considering a wide range of inlet velocities (*u*_0_), while keeping *T*_go_ = 298 K, *P* = 1 atm, and Ø = 0.5 constant, a global energy balance was applied to the burner (see Figure 3). From this analysis, the energy contributions associated with the thermal power, radiative heat transfer, and the energy carried by the exhaust gases were identified. It should be noted that, in the numerical analysis, heat losses through the walls were neglected.

The numerical results are represented by solid lines in Figure 8 and correspond to the idealized upper-performance limit predicted by the thermodynamic model. The experimental data are shown as markers for comparison. The experimental energy terms associated with the thermal power input (gray markers), the energy carried by the exhaust gases (yellow markers), and the radiative heat transfer from the burner outlet surface (red markers) were determined from measurements of the air and fuel mass flow rates and the outlet surface temperature (black markers).

**Figure 8:**
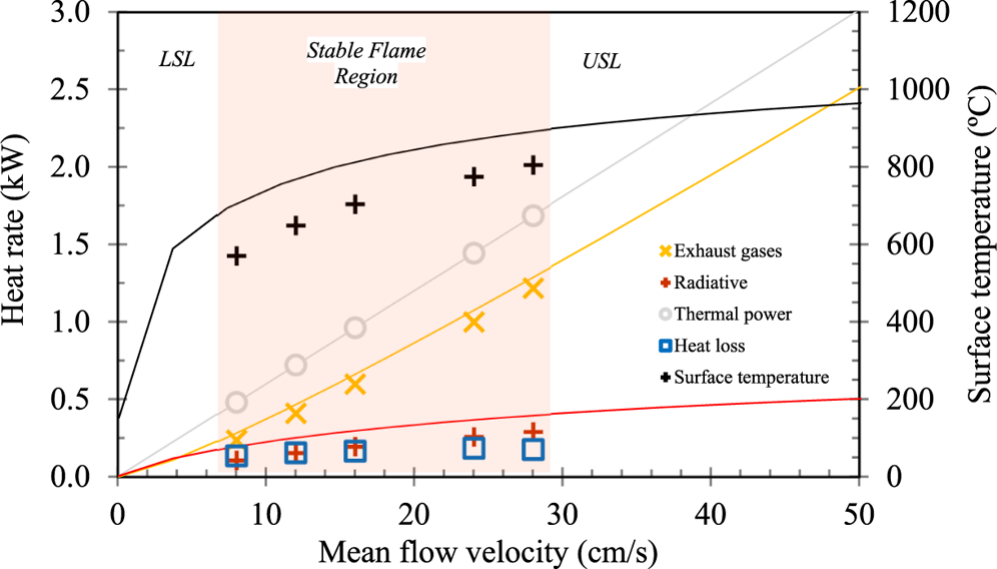
Numerical (solid lines) and experimental values (markers) for the radiative heat transfer rates (red), exhaust gases energy transport (yellow), thermal power (gray), heat loss (blue) and surface temperature (black) for a 50% CO_2_ + 50% CH_4_ mixture with preheating and Ø = 0.5.

Based on these energy terms, the experimental heat losses (blue markers) were estimated through an overall energy balance. The comparison between numerical and experimental results is intended to assess whether the predicted energy-transfer trends are consistent with the observed burner behavior and to quantify the deviation between the idealized performance limit and the actual burner performance. The red shaded area represents the flame stability region experimentally obtained with the coupled heat exchanger for the fuel mixture containing 50% CO_2_ + 50% CH_4_.

The numerical results (solid lines) show that thermal power increases linearly with mean flow velocity due to the higher fuel mass flow rate, while the equivalence ratio remains constant. Since the model used the same inlet conditions as the experiments, the thermal power values are identical in both approaches.

The predicted reaction temperature represents the theoretical maximum, assuming negligible heat losses, complete combustion, and thermal equilibrium between gas and solid phases. Consequently, experimental temperatures are lower and at *u*_0_ = 8 cm/s, the surface temperature difference was about 20%.

For 0 < *u*_0_ < 10 cm/s, increasing flow velocity causes a sharp temperature rise, followed by stabilization at higher velocities. The initial rise enhances radiative heat transfer and exhaust gas energy. However, for *u*_0_ > 10 cm/s, further velocity increases mainly intensify energy carried by the exhaust gases, as temperature growth becomes marginal and radiation strongly depends on temperature.

Experimentally, the stability range occurs after this sharp temperature increase, where higher flow velocity primarily raises thermal power, while radiative heat transfer grows less significantly, indicating the dominant role of exhaust gas energy in reducing radiative efficiency. Measured heat losses, due to wall heat transfer, gas–solid thermal nonequilibrium, and incomplete combustion, led to lower outlet surface temperatures and ranged from 13 to 17% across the stability region.

### 3.4 Pollutant Emission Index

Figure 9 presents the CO emission index as a function of the mean flow velocity. Results are shown for Ø = 0.50 with varying CH_4_ concentrations (vol %), under preheated (red markers) and nonpreheated (black markers) conditions.

**Figure 9:**
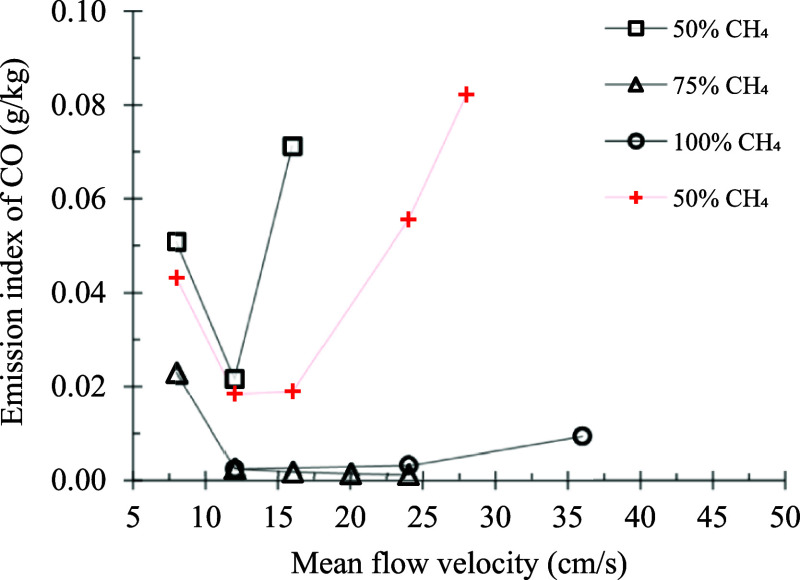
Emission index of CO as a function of the mean flow velocity for different fuel mixtures at Ø = 0.50, with (red markers) and without (black markers) reactant preheating.

It can be observed that lower flow velocities result in higher CO emission index. This behavior is associated with the lower temperatures (<1000 °C) achieved within the porous medium, which limit CO oxidation and hinder CO_2_ formation through the reaction CO + OH ↔ CO_2_ + H.^15^ As the flow velocity increases, the CO emission index initially decreases, reaching a minimum value, and subsequently increases again due to the reduced gas residence time in the porous medium.^33^

The influence of flame temperature on CO oxidation was further evidenced in the analysis of the effects of CO_2_ and CH_4_ concentrations in the biogas on pollutant emissions. For a flow velocity of 12 cm/s and without reactant preheating (black markers), increasing the CO_2_ concentration from 0 to 50% resulted in higher CO emission index, due to the reduction in reaction-zone temperature. Conversely, for the same flow velocity with reactant preheating (red markers), the higher inlet temperature increased the reaction-zone temperature and promoted CO oxidation, leading to lower CO emissions. The maximum CO emission index measured under all operating conditions evaluated in the present study was 0.082 g/kg.

NO emission index remained below the measurement uncertainty of the analyzer for all tested conditions and were therefore considered negligible.

### 3.5 Numerical Analysis of Adiabatic Laminar Flame Speed and Flame Temperature

The effects of fuel mixture composition and reactant preheating on flame behavior in the porous medium are directly related to the adiabatic flame speed (*S*_L0_) and adiabatic flame temperature (*T*_ad_). Thus, the chemical kinetics of the studied mixtures were numerically evaluated under the same experimental operating conditions using CHEMKIN 2025. The GRI-Mech 3.0 mechanism^34^ was applied with grid refinement parameters of grad = 0.01 and curve = 0.05, using a 10,000-point mesh. The GRI-Mech 3.0 mechanism was selected due to its extensive validation for methane combustion and its demonstrated applicability to biogas mixtures, with previous studies supporting its viability for simulating CH_4_/CO_2_-based fuels.^35^

Figure 10a presents the adiabatic flame speed as a function of fuel mixture at Ø = 0.5 and *P* = 1 atm, for cases with (red markers) and without (black markers) reactant preheating. For preheated conditions, *S*_L0_ was calculated for 50% CH_4_ + 50% CO_2_ and 100% CH_4_ mixtures, using experimentally measured reactant temperatures at 24 cm/s: 111.2 and 142.5 °C, respectively (Table 4).

Figure 10b shows the adiabatic flame temperature versus the dimensionless axial distance in the one-dimensional flame domain for all mixtures at Ø = 0.5 and *P* = 1 atm, with (red lines) and without (black lines) reactant preheating.

Analyzing the condition without reactant preheating (Figure 10a), the reduction in CH_4_ concentration from 100% to 35% leads to a decrease in *S*_L0_ from 4.7 to 1.8 cm/s, respectively. This reduction is associated with the thermal and chemical effects of carbon dioxide, including the increase in the mixture heat capacity, the decrease in the adiabatic flame temperature, and the dilution of reactive radicals, which collectively slow down the overall reaction rates.^36,37^

**Figure 10:**
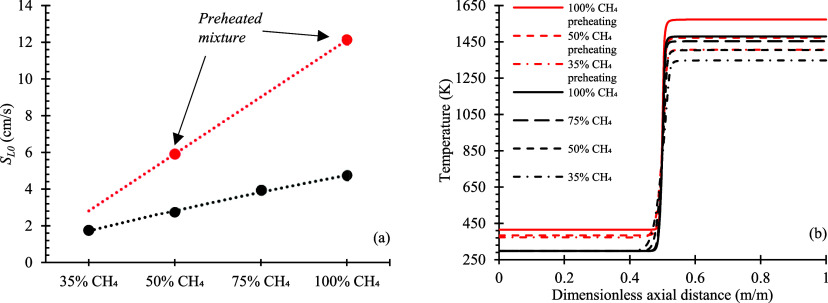
Numerical predictions of (a) adiabatic laminar flame speed (*S*_L0_) as a function of the fuel mixture and (b) adiabatic flame temperature as a function of the dimensionless axial distance, for all fuel mixtures at Ø = 0.5 and *P* = 1 atm, with (red markers) and without (black markers) reactant preheating.

When reactant preheating is considered, an increase of approximately 152% in the laminar flame speed is observed for pure methane, reaching a value of about 12 cm/s. For the mixture containing 50% CH_4_, the laminar flame speed increased by approximately 118% compared to the nonpreheated condition. These differences in adiabatic flame speed due to preheating result from both the fuel mixture composition and the different preheating temperatures experimentally achieved under the same mean flow velocity of 24 cm/s. Furthermore, reactant preheating enhances chain-branching reactions and accelerates the global combustion kinetics, leading to higher *S*_L0_ values even for highly diluted mixtures.^36,38^

Regarding the adiabatic flame temperature (Figure 10b) it can be observed that reducing the CH_4_ concentration from 100% to 35% decreases the temperature from 1480 K (1207 °C) to 1347 K (1074 °C), corresponding to a percentage reduction of about 9%. Additionally, for the mixtures containing 50% and 100% CH_4_, the use of the heat exchanger increased the adiabatic flame temperature by approximately 6%.

## 4 Conclusions

In this study, a radiant porous burner with an integrated heat exchanger was experimentally and numerically evaluated using CH_4_ + CO_2_ mixtures to represent different biogas compositions. Stable operation was achieved up to 65 vol % CO_2_, despite the significant reduction in lower heating value. Higher dilution caused flame instability and extinction, defining the flammability limit under the tested conditions. Although uncertainties in equivalence ratio and flow velocity may influence the exact location of the stability boundaries, the overall stability trends identified in this study remain consistent within the estimated measurement uncertainty. The reported stability limits were established according to the experimental criteria adopted in this work. A quantitative assessment of the repeatability of the stability boundaries could be addressed in future studies through dedicated repeated measurements performed in the vicinity of the stability limits.

Increasing CO_2_ concentration reduced the reaction zone temperature and shifted the flame front toward the burner outlet, while the surface temperature remained nearly constant. Although reactant preheating was associated with a reduction in the estimated heat losses, the measured radiative efficiency remained approximately constant within the experimental uncertainty. Therefore, no statistically significant variation in radiative efficiency could be established under the investigated conditions.

Reactant preheating significantly expanded the stability range. For the 50% CH_4_ + 50% CO_2_ mixture, the upper stability limit increased from 16 to 28 cm/s (∼75% expansion). This improvement resulted from higher reaction zone temperatures, which increased laminar flame speed and stabilized the flame at the porous layer interface. Although reactant preheating increased the temperature near the burner base, the adopted flashback criterion was not reached under the investigated conditions. Therefore, no measurable shift of the lower stability limit was observed experimentally. Nevertheless, a physical influence of preheating on the lower stability limit cannot be excluded, since the increase in mixture reactivity and flame speed may promote upstream flame propagation. Kinetic simulations with GRI-Mech 3.0 confirmed that preheating raised the adiabatic flame speed by ∼118%, directly contributing to the wider stability range.

It should be noted that the thermodynamic model provides an idealized upper limit, and deviations from experimental results are expected due to heat losses, gas–solid thermal nonequilibrium, and nonideal combustion effects. As a result, the model overpredicts outlet surface temperatures and radiative efficiency compared to experimental measurements. The global energy balance indicated heat losses in the range of 13–17%, which explains the observed discrepancies under practical operating conditions. Within the stable regime, increasing flow velocity primarily intensified the energy carried by the exhaust gases rather than radiative heat transfer, highlighting exhaust-related losses as a key performance limitation.

Preheating also promoted cleaner combustion, with a maximum CO emission index of 0.082 g kg^–1^ and NO emissions below detection limits. Overall, the combined effects of CO_2_ dilution and reactant preheating provide valuable insights for efficient use of low-calorific biogas in radiant porous combustion systems.
